# Which type of congenital malformations is significantly increased in singleton pregnancies following after *in vitro* fertilization/intracytoplasmic sperm injection: a systematic review and meta-analysis

**DOI:** 10.18632/oncotarget.23689

**Published:** 2017-12-25

**Authors:** Ying Liang, Letao Chen, Hong Yu, Hua Wang, Qi Li, Renhe Yu, Jiabi Qin

**Affiliations:** ^1^ Department of Epidemiology and Health Statistics, Xiangya School of Public Health, Central South University, Hunan, China; ^2^ Hunan Provincial Maternal and Child Health Hospital, Hunan, China

**Keywords:** IVF/ICSI, specific congenital malformations, cohort study, meta-analysis, singleton pregnancies

## Abstract

It is inconclusive nowadays for which type of congenital malformations(CMs) is increased in singleton pregnancies following after *in vitro* fertilization(IVF)/intracytoplasmic sperm injection(ICSI) compared with those after spontaneous conception; furthermore, a complete overview is missing. We conducted a meta-analysis of cohort studies to assess the risk of specific CMs associated with IVF/ICSI singleton pregnancies. Unrestricted searches were conducted, with an end date parameter of 1 June 2017, of PubMed, Embase, Google Scholar, Cochrane Libraries, and Chinese databases. Either a fixed- or a random-effects model was used to calculate the overall combined risk estimates. Subgroup and sensitivity analyses were performed to explore potential heterogeneity moderators when significant heterogeneity was observed. Sixteen cohort studies with a total of 129,648 IVF/ICSI and 5,491,949 spontaneously conceived singleton births fulfilled the inclusion criteria. The IVF/ICSI singleton pregnancies had a significantly increased risk of cleft lip and/or palate (OR = 1.34 [95% CI: 1.07–1.69]; *I* ^2^ = 0%), eye, ear, face and neck (odd ratios [OR] = 1.20 [95% CI: 1.04–1.39]; *I* ^2^ = 15%), chromosomal (OR = 1.23 [95% CI: 1.07–1.40]; *I* ^2^ = 32%), respiratory (OR = 1.28 [95% CI: 1.01–1.64]; *I* ^2^ = 37%), digestive (OR = 1.46 [95% CI: 1.29–1.65]; *I* ^2^ = 0%), musculoskeletal (OR = 1.47 [95% CI: 1.25–1.72]; *I* ^2^ = 64%), urogenital (OR = 1.43 [95% CI: 1.18–1.72]; *I* ^2^ = 62%), and circulatory (OR = 1.39 [95% CI: 1.23–1.58]; *I* ^2^ = 46%) system malformations. Relevant heterogeneity moderators have been identified by subgroup analysis. Sensitivity analysis yielded consistent results. No evidence of publication bias was observed. In conclusion, the IVF/ICSI singleton pregnancies are associated with higher risks for most specific CMs. Clinicians should provide appropriate information to counseling IVF/ICSI patients.

## INTRODUCTION

Since the birth of the first infant conceived with *in vitro* fertilization (IVF) in the United Kingdom in 1978, the number of pregnancies and births resulting from assisted reproductive technologies (ART) has been increasing [1], with an estimated 200,000 ART births worldwide each year [2–3]. To date, this technology has resulted in more than 5 million infants born globally [4]. Although this technology is generally considered safe, its impact on the health of children did not experience formal evaluation when it was introduced into practice [5]. In consideration of rapidly growing population using this technology, it is very important to continually monitor the safety of ART.

A large number of cohort studies [2, 5–12] and meta-analyses [13–20] have affirmed that children conceived with ART involving IVF and/or intracytoplasmic sperm injection (ICSI) compared with those spontaneously conceived (SC) had a significantly increased risk of developing congenital malformations (CMs). It seems to be stronger for singleton births than for multiple births for the associations between ART procedures and CMs [9, 14, 20]. Our previous two reviews [21–22] also indicated that the risk of CMs was remarkably increased in the IVF and/or ICSI singleton pregnancies. However, it is still unknown for the risk of specific CMs associated with singleton pregnancies generated by ART. With a wide implementation of single-embryo transfer (SET) in many countries [23], it is very necessary to resolve this issue. Although many epidemiologic studies [2, 5–12, 24–30] have been conducted to investigate the link between ART and risk of specific CMs in singleton pregnancies, the magnitudes of the association varied between studies and the results are often inconsistent. Furthermore, a complete overview is missing.

An improved understanding of this issue may have important clinical implication considering the fact that the evaluation of risk for the specific CMs is a fundamental step for an adequate pre-conception counseling for infertile couples who selected ART to achieve a pregnancy [31]. Most of available studies have been limited by small numbers of participants [9], and sufficiently powered studies are needed to evaluate associations, particularly with regard to specific CMs. Therefore, we conducted a systematic review and meta-analysis of cohort studies to address whether singleton pregnancies following after IVF and/or ICSI have a significantly higher risk of specific CMs compared with those conceived naturally.

## RESULTS

### Search results and study selection

**Figure 1:**
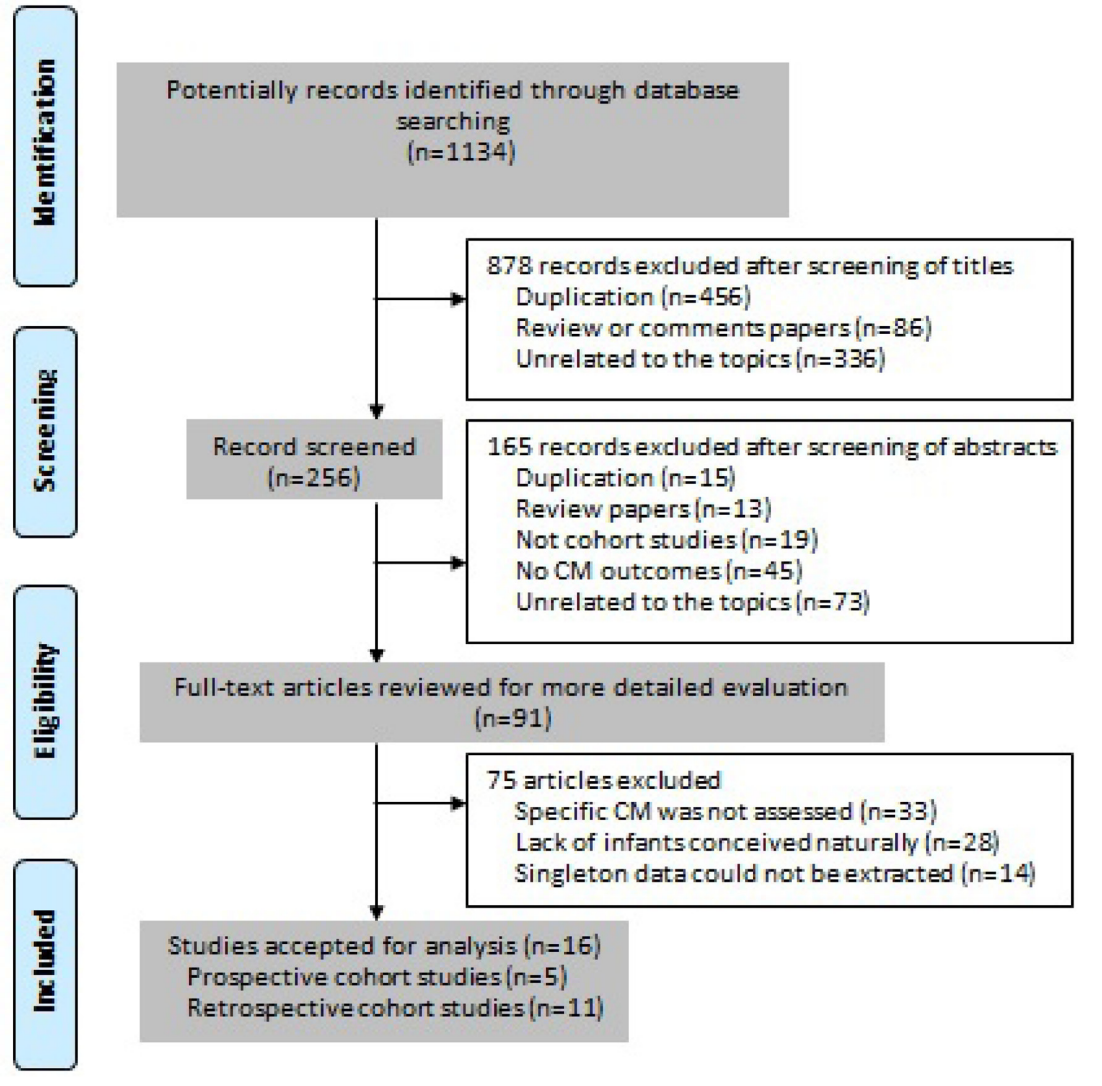
PRISMA flow chart

After the computerized search in eight databases, total 1,134 records were initially identified. We then found 91 articles to be potentially eligible after reading the title or abstract, of which 16 studies [2, 5–12, 24–30] fulfilled the inclusion criteria (Figure 1). Reasons for not including the other studies were: (i) specific CM was not assessed (*n* = 33); (ii) lack of control group of infants conceived naturally (*n* = 28); and (iii) singleton data could not be extracted (*n* = 14).

### Characteristics of included studies

The characteristics of included studies including 129,648 singleton births conceived with ART as well as 5,491,949 singleton births conceived spontaneously, were summarized in Table 1. Seven studies [7, 8, 10, 24–26, 29] were conducted in Europe, 4 [2, 5, 6, 9] in Australia, 3 [11, 12, 28] in the United States, 1 [30] in Canada, and 1 [27] in Japan. Ten studies (62.5%) did not include patients who achieved a pregnancy with OI or IUI in the SC group, but the remaining studies (37.5%) included these patients in the SC group.

**Table 1:** Characteristics of the included studies

First author/ publication year (study period)	Geographic region	Sample sourcea	Study design	ART singleton births (*n*)	SC singleton births (*n*)	Type of ART	Whether patients who achieved a pregnancy with OI and IUI were included in the SC group?	Reported specific CM	Diagnostic age or time of CMs	Adjusted, matched or crude data	Quality scoreb
Dhont M/1997^*^ (1991–1995)	Belgium	Population	retrospective cohort	311	622	IVF/ICSI	no	Circulatory system; Chromosomal defects; Urogenital system	Birth	*Matched*	1
Isaksson R/2002^*^ (1993–1999)	Finland	Clinic	retrospective cohort	1901	345	IVF/ICSI	no	Urogenital system; Chromosomal defects	Birth	Matched	2
Hansen M/2002 (1993–1997)	Australia	Clinic	prospective cohort	713	3906	IVF; ICSI	no	Musculoskeletal system; Urogenital system; Chromosomal defects; Digestive system; Circulatory system; Central nervous system	12 months	Crude	1
Bonduelle M/2005 (2000–2005)	Five European countries^	Population	prospective cohort	977	538	IVF; ICSI	no	Musculoskeletal system; Urogenital system; Eye, ear, face and neck; Digestive system; Circulatory system	5 years	Matched	1
Olson CK/2005 (1989–2002)	USA	Clinic	retrospective cohort	645	4590	IVF	no	Respiratory system; Musculoskeletal system; Urogenital system; Eye, ear, face and neck; Chromosomal defects; Digestive system; Circulatory system; Central nervous system	12 months	Matched	1
Klemetti R/2005 (1996–1999)	Finland	Population	prospective cohort	2930	26489	IVF	no	Cleft lip and/or palate; Respiratory system; Musculoskeletal system; Urogenital system; Eye, ear, face and neck; Chromosomal defects; Digestive system; Circulatory system; Central nervous system	1 year	Crude	1
Pinborg A/2010 (1995–2007)	Denmark	Population	prospective cohort	11453	4800	IVF; ICSI	yes	Respiratory system; Musculoskeletal system; Urogenital system; Eye, ear, face and neck; Chromosomal defects; Digestive system; Circulatory system; Central nervous system	1–13 years	Crude	1
Wen SW/2010* (1996–2005)	Canada	Clinic	retrospective cohort	568	1100	IVF/ICSI	no	Musculoskeletal system; Chromosomal defects; Digestive system; Circulatory system	Birth	Adjusted and matched	1
Halliday JL/2010 (1991–2004)	Australia	Population	retrospective cohort	6946	20838	IVF;ICSI	yes	Musculoskeletal system; Urogenital system; Chromosomal defects; Circulatory system	Birth	Adjusted	1
Hansen M/2012^*^ (1994–2002)	Australia	Population	retrospective cohort	1972	205641	IVF/ICSI	yes	Musculoskeletal system; Urogenital system; Chromosomal defects; Digestive system; Circulatory system; Central nervous system	6 years	Adjusted	1
Sagot P/2012 (2000–2009)	France	Population	retrospective cohort	903	4044	IVF	no	Cleft lip and/or palate; Respiratory system; Musculoskeletal system; Urogenital system; Eye, ear, face and neck; Chromosomal defects; Digestive system; Circulatory system; Central nervous system	Birth	Adjusted and matched	1
Davies MJ/2012^*^ (1986–2002)	Australia	Population	retrospective cohort	4333	295220	IVF/ICSI	no	Respiratory system; Musculoskeletal system; Urogenital system; Chromosomal defects; Digestive system; Circulatory system; Central nervous system	5 years	Adjusted	1
Fedder J/2013 (1995–2009)	Denmark	Population	prospective cohort	17216	33852	IVF; ICSI	yes	Respiratory system; Musculoskeletal system; Urogenital system; Eye, ear, face and neck; Chromosomal defects; Digestive system; Circulatory system; Central nervous system	Birth	Crude	1
Jwa J/2015^*^ (2010–2012)	Japan	Population	retrospective cohort	34949	36157	IVF/ICSI	yes	Respiratory system; Musculoskeletal system; Urogenital system; Eye, ear, face and neck; Chromosomal defects; Digestive system; Circulatory system; Central nervous system	Birth	Adjusted	1
Boulet SL/2016^*^ (2000–2010)	USA	Population	retrospective cohort	33601	4421154	IVF/ICSI	yes	Cleft lip and/or palate; Chromosomal defects; Digestive system; Circulatory system; Central nervous system	1 year	Adjusted	1
Liberman RF/2017^*^ (2004–2011)	USA	Population	retrospective cohor	10230	432653	IVF/ICSI	no	Circulatory system; Central nervous system; Respiratory system; Eye, ear, face and neck; Cleft lip and/or palate; Digestive system; Urogenital system; Musculoskeletal system	1 year	Adjusted	1

Abbreviations: ART: assisted reproductive technology; SC: spontaneously conceived; IUI: intrauterine insemination; OI: ovulation induction; ICSI: intracytoplasmic sperm injection; IVF: *in vitro.* fertilization; CM: congenital malformations.

^a^Population versus clinic-based sample.

^b^Each study was assigned a score out of 9; 1 = higher quality studies with scores ≥7, 2 = low quality with scores <7.

^^^UK, Belgium, Sweden, Denmark, and Greece.

^*^These papers did not estimate obstetric risks in IVF and ICSI pregnancies separately.

Fifteen studies (93.8%) were considered to be of high quality; these 15 studies contributed 98.5% of the ART births and 100.0% of the SC births. Only four studies [5, 7, 8, 10] did not adjust and/or match any factors when estimating the risk of specific CMs associated with ART. Total number of ART and SC singleton births involved for each specific CMs is summarized in Supplementary Table 1.

### ART and risk of cleft lip and/or palate, eye, ear, face and neck, and nervous system malformations

**Figure 2:**
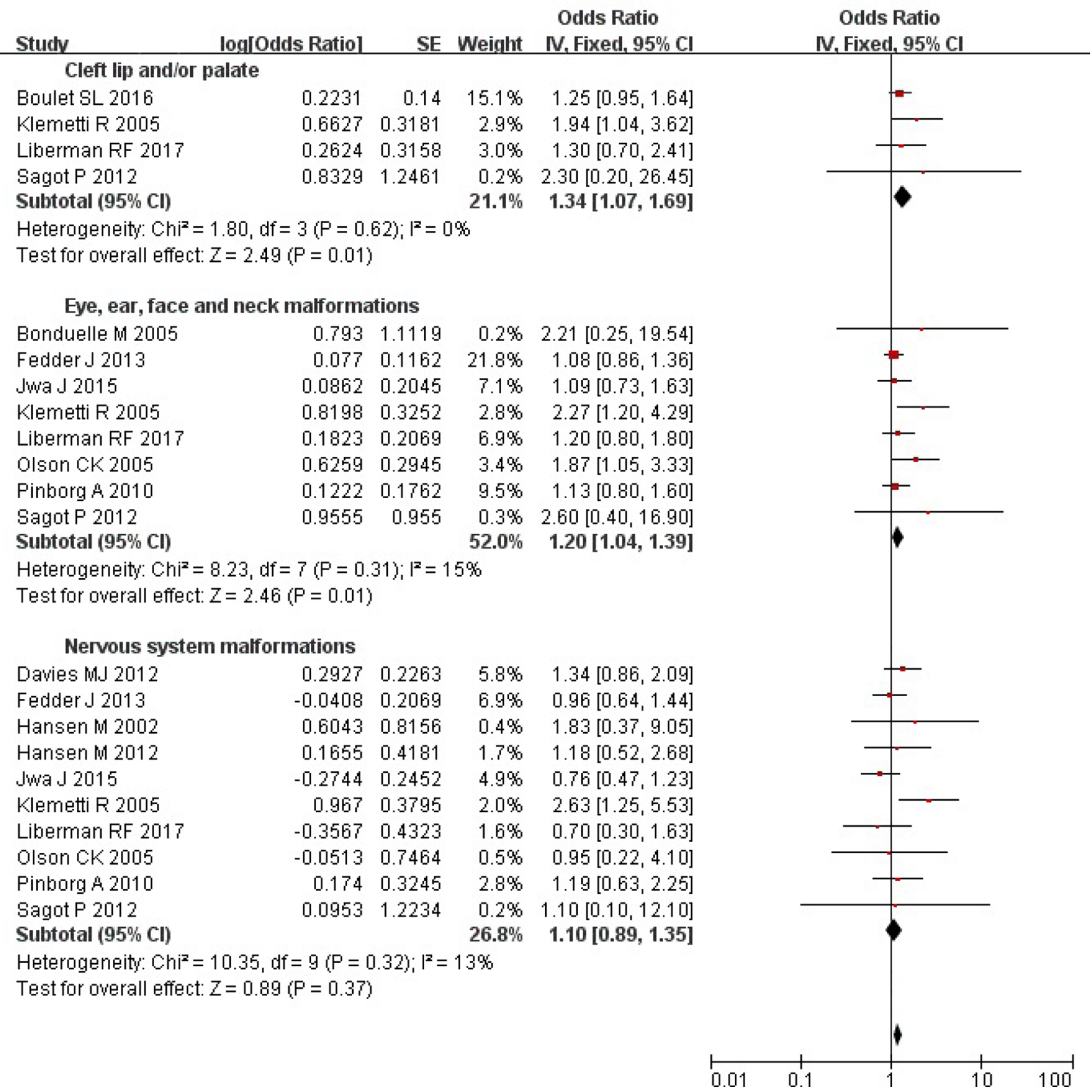
Forest plot for ART and risk of cleft lip and/or palate, eye, ear, face and neck, and nervous system malformations in singleton pregnancies

Overall, the ART singleton pregnancies compared with those conceived spontaneously had a higher risk of cleft lip and/or palate (OR = 1.34; 95% CI: 1.07–1.69), and eye, ear, face and neck malformations (OR = 1.20; 95% CI: 1.04–1.39), yet there was not a significantly higher risk of nervous system malformations (OR = 1.10; 95% CI: 0.89–1.35) (Figure 2). We did not find the evidence of heterogeneity (all *P* values ≥ 0.31; all *I* ^2^ values ≤ 15%). Additionally, the random-effects model yielded similar results.

### ART and risk of chromosomal, respiratory and digestive system malformations

**Figure 3:**
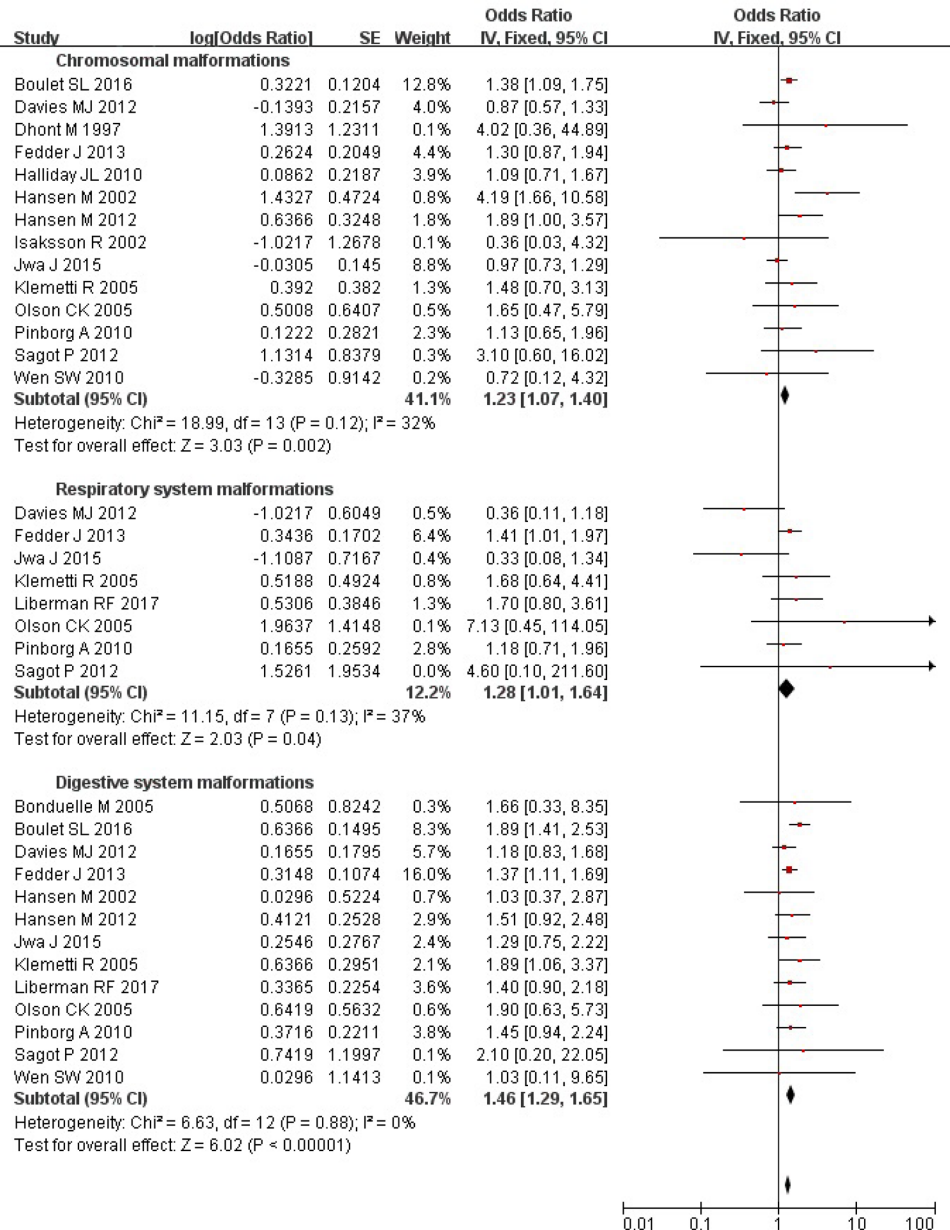
Forest plot for ART and risk of chromosomal, respiratory and digestive system malformations in singleton pregnancies

Overall, the ART singleton pregnancies were at a significantly increased risk of chromosomal (OR = 1.23; 95% CI: 1.07–1.40), respiratory (OR = 1.28; 95% CI: 1.01–1.64), and digestive (OR = 1.46; 95% CI: 1.29–1.65) system malformations (Figure 3). Substantial heterogeneity was not observed (all *P* values ≥ 0.12; all *I* ^2^ values ≤ 37%). Further analysis using the random-effects model brought consistent results except for respiratory system malformations (OR = 1.20; 95% CI: 0.82–1.77).

### ART and risk of musculoskeletal, urogenital, and circulatory system malformations

**Figure 4:**
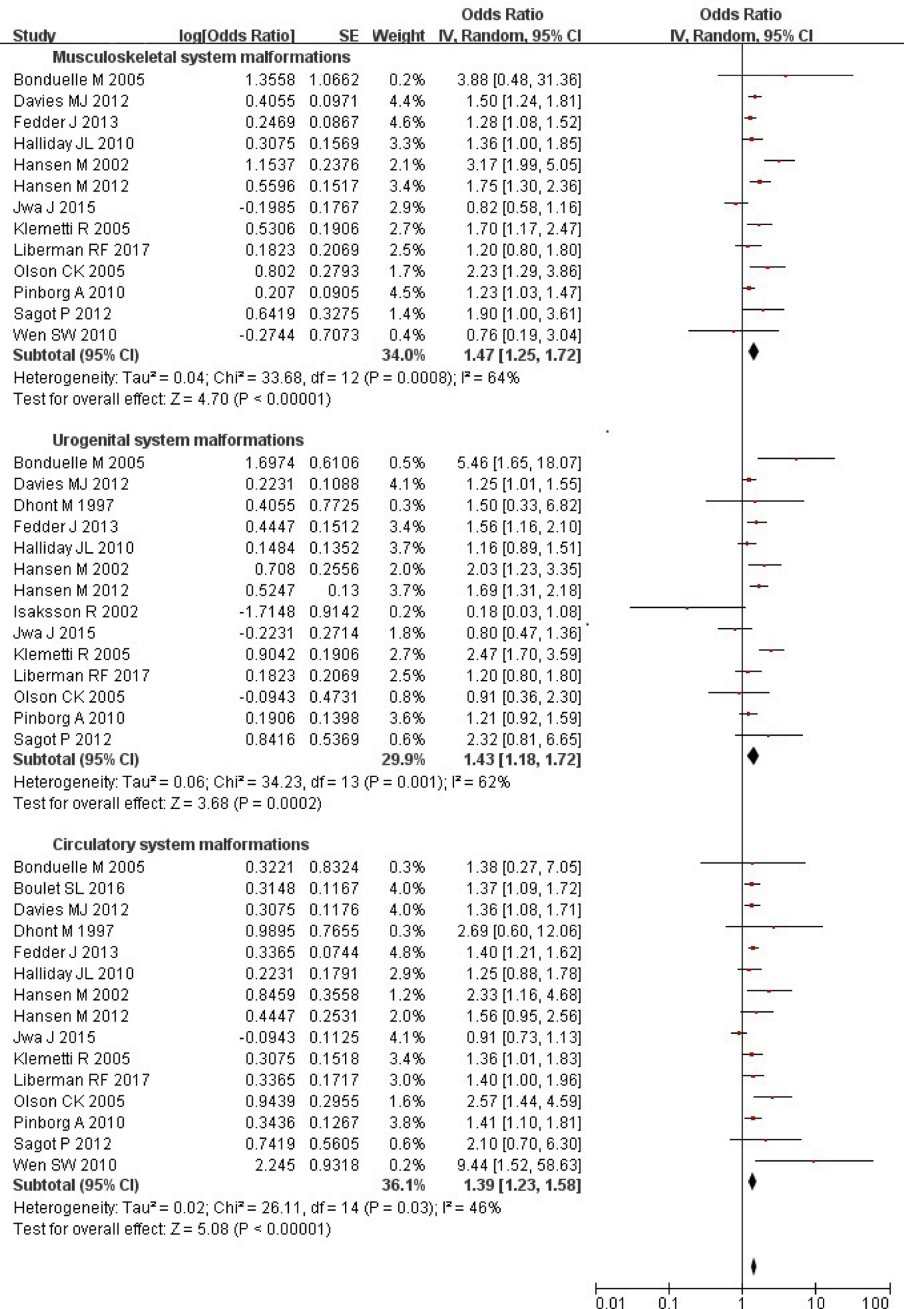
Forest plot for ART and risk of musculoskeletal, urogenital, and circulatory system malformations in singleton pregnancies

Overall, the risks of musculoskeletal (OR = 1.47; 95% CI: 1.25–1.72), urogenital (OR = 1.43; 95% CI: 1.18–1.72), and circulatory (OR = 1.39; 95% CI: 1.23–1.58) system malformations were evidently higher in the ART-conceived births than those conceived spontaneously (Figure 4). Substantial heterogeneity was observed (all *P* values ≤ 0.03; all *I* ^2^ values ≥ 46%). The results were similar after further analysis using the fixed-effects model.

### Subgroup analysis

Subgroup analysis for all specific malformations in singleton pregnancies was summarized in Supplementary Table 2. After subgroup analysis, whether the SC group included women who used OI and IUI to get a pregnancy, geographic regions, quality scores, sample sources, and diagnostic age or time of CMs were identified as the most relevant heterogeneity moderators. Risks of musculoskeletal system malformations in singleton pregnancies generated by ART were significantly different for whether the SC group included women who used OI and IUI to get a pregnancy (test for subgroup differences [TSD]: χ^2^ = 7.90; *P* = 0.02), geographic regions (TSD: χ^2^ = 11.31; *P* = 0.01), and diagnostic age or time of CMs (TSD: χ^2^ = 4.66; *P* = 0.03). There were statistically significant differences for risk of circulatory system malformations in ART singleton pregnancies across different sample sources (TSD: χ^2^ = 9.74; *P* = 0.002) and geographic regions (TSD: χ^2^ = 14.52; *P* = 0.002). Besides, the risk was significantly different for urogenital system malformations across quality scores (TSD: χ^2^ = 5.26; *P* = 0.02), for eye, ear, face and neck malformations for whether the SC group included women who used OI and IUI to get a pregnancy (TSD: χ^2^ = 7.53, *P* = 0.02), and for nervous system malformations across diagnostic age or time of CMs (TSD: χ^2^ = 5.05; *P* = 0.02).

### Sensitivity analysis

When evaluating the risk of musculoskeletal (*P* = 0.0008; *I* ^2^ = 64%), urogenital (*P* = 0.001; *I* ^2^ = 62%), and circulatory (*P* = 0.03; *I* ^2^ = 46%) system malformations associated with ART, we found substantial heterogeneity. So we used sensitivity analysis to explore potential sources of heterogeneity. Exclusion of 4 studies [5, 7, 8, 10] in which the confounding factors were not adjusted or matched yielded similar results(OR = 1.41 [95% CI: 1.15–1.74], 1.28 [95% CI: 1.01–1.62], and 1.40 [95% CI: 1.15–1.71] for musculoskeletal, urogenital, and circulatory system malformations, respectively), with substantial evidence of heterogeneity (all *P* values ≤ 0.02; all *I* ^2^ values ≥ 55%). Exclusion of 6 studies [2, 6, 8, 10, 11, 27] in which the SC group included women who used OI and IUI to get a pregnancy suggested a somewhat greater risk (OR = 1.76 [95% CI: 1.37–2.25], 1.59 [95% CI: 1.12–2.25], and 1.60 [95% CI: 1.30–1.96] for musculoskeletal, urogenital, and circulatory system malformations, respectively), yet there was still substantial evidence of heterogeneity for musculoskeletal (*P* = 0.05; *I* ^2^ = 50%) and urogenital (*P* = 0.002; *I* ^2^ = 66%) system malformations. Furthermore, the results were consistent after exclusion of any one study at a time.

### Publication bias

Begg funnel plot (Supplementary Figure 1) and Egger’s regression test (*P* = 0.6048, 0.8031, 0.7096, 0.5080, 0.3048, 0.6003, 0.1239, 0.2984, and 0.1117 for cleft lip and/or palate, eye, ear, face and neck, nervous, chromosomal, respiratory, digestive, musculoskeletal, urogenital, and circulatory system malformations, respectively) showed there was no evidence of publication bias.

## DISCUSSION

Finding from present study indicated that the singleton pregnancies created with ART, compared with those after spontaneous conception, experienced a significantly increased risk of 34% for cleft lip and/or palate, 20% for eye, ear, face and neck malformations, 23% for chromosomal defects, 28% for respiratory system malformations, 46% for digestive system malformations, 47% for musculoskeletal system malformations, 43% for urogenital system malformations, and 39% for circulatory system malformations, without significantly increasing the risk of nervous system malformations (*P* = 0.37). Substantial heterogeneity was not found for most outcomes except for musculoskeletal, urogenital, and circulatory system malformations.

In general, based on available data, we know that the ART singleton births have been found to be associated with an increase in CMs, many studies of which are now summarized in systematic reviews [13–21]. What is unknown, however, is whether singleton births resulting from ART compared with those conceived spontaneously had a higher risk of specific CMs. Unlike past reviews, the present study only focused on the specific CMs after ART singleton pregnancies because the risk of overall CMs have been confirmed by past meta-analysis [15–16, 18–21]. By using a classification system, which groups CMs into body systems, we found a specific association between ART and specific CMs.

Although some cohort studies have been conducted to explore the risk of specific CMs associated with IVF/ICSI procedures in singleton pregnancies, the results are often inconsistent. For example, Klemetti *et al.* [7] reported a significantly increased risk of cleft lip and/or palate, eye, ear, face and neck, digestive, musculoskeletal, urogenital, circulatory, and nervous system malformations in the IVF/ICSI singleton pregnancies than the reference group; Hansen *et al.* [5–6], Sagot *et al.* [29], and Davies *et al.* [9] reported a higher risk of chromosomal, musculoskeletal, urogenital, and circulatory system malformations; Boulet *et al.* [11] indicated an increased risk of chromosomal, digestive and circulatory system malformations; Liberman *et al.* [12] only found an increased risk of circulatory system malformations; yet other studies did not find statistically significant differences between ART and SC singleton pregnancies for the risk of most of specific CMs. Our review has enhanced statistical power to provide more precise and reliable risk estimates by collecting published studies.

In the present study, the risk of most specific CMs was evidently increased in the ART singleton pregnancies, but the reasons are not clear, which was rarely discussed in previous studies. A possible explanation is that the IVF/ICSI procedures themselves or maternal factors associated with infertility or a combination of these factors brought about increased risks of CMs in the IVF/ICSI pregnancies [32, 33]. For example, it has been discussed that factors associated with ART procedures themselves such as the medications used to induce ovulation or to maintain the pregnancy in the early stages, the culture media composition, the length of time in culture, the freezing and thawing of embryos, the potential for polyspermic fertilization, the delayed fertilization of the oocyte, altered hormonal environment at the time of implantation, the manipulation of gametes and embryos or a combination of these may increase the risk of CMs [16, 33].

Additionally, some studies [9, 29, 34–35] shown that infertility itself including older age of infertile couples and their underlying infertility factors may also bring an increased risk of specific CMs. Our previous cohort study [33] comparing the obstetric outcomes of women treated with IVF, women with indicators of subfertility but without IVF/ICSI, and fertile women with singleton pregnancies confirmed an increased risk of CMs in IVF pregnancies may be a result of the IVF procedures themselves and the infertility itself together. However, the underlying mechanisms between ART and risk of specific CMs are still unclear.

Substantial heterogeneity was observed when we evaluated the risk of musculoskeletal (*P* = 0.0008; *I* ^2^ = 64%), urogenital (*P* = 0.001; *I* ^2^ = 62%), and circulatory (*P* = 0.03; *I* ^2^ = 46%) system malformations in the ART singleton pregnancies. In our review, subgroup analysis has identified main heterogeneity moderators, such as diagnosis time of CMs, geographic region, whether the SC group included women who used OI and IUI to get a pregnancy, quality scores, and sample sources. Some authors considered that ideally case and control infant should be assessed no earlier than at the age of 6 months, as it has been reported that 90% of major malformations are diagnosed by the age of 6 months and only 66% at birth [19]. In the present review, more than half (56.3%) of included studies has monitored the occurrence of CMs in the offspring at least 1 year after birth. Our study also showed a significant difference for risk of musculoskeletal and circulatory system malformations in different geographic regions and whether the SC group included women who used OI and IUI to get a pregnancy, which was supported by previous studies [29, 32, 35]. Although there was still evidence of heterogeneity after subgroup or sensitivity analyses, the result with very few changes was stable and reliable.

The present study represents, to our knowledge, the first meta-analysis of specific CMs risks associated with ART singleton pregnancies. Although some studies have been conducted to investigate the link between ART and risk of specific CMs in singleton pregnancies, the results are often inconsistent. With the accumulating evidence and enlarged sample size, we have enhanced statistical power to provide more precise and reliable risk estimates. However, potential limitations should be considered in future studies. First, the classification of CMs was different across studies, which may lead to classification bias. Second, there was substantial heterogeneity among studies for association between ART and some specific CMs. Although we were able to detect the major source of heterogeneity through the subgroup analysis and the sensitivity analysis, Our results should be treated with caution because of heterogeneity. Third, the number of included studies is limited for a number of specific CMs, so more studies should be included in future reviews, to provide further support for our results. Finally, because the present review only included studies published in Chinese or English, additional research in other populations is warranted to generalize the findings.

In conclusion, our results showed that cleft lip and/or palate, eye, ear, face and neck, chromosomal, respiratory, digestive, musculoskeletal, urogenital, and circulatory system malformations were the most reported CMs associated with ART singleton pregnancies according to organs and systems classification. With the increasing implementation of SET, an improved understanding of this issue may have important clinical implications, given the possibility that the clear results might be useful for counseling ART patients and properly designing the consent forms. However, the mechanisms between ART and specific CMs remain unclear and require further study for elucidation.

## MATERIALS AND METHODS

We followed the recommendations of the Preferred Reporting Items for Systematic Reviews and Meta-analyses (PRISMA) statement [36] to report the present systematic review and meta-analysis. The research protocol for the present meta-analysis has passed the PROSPERO registration (registration number: CRD42017071802).

### Search strategy

Relevant studies assessing risk of specific CMs in the ART singleton pregnancies compared with those conceived naturally were identified. Unrestricted searches were conducted, with an end date parameter of 1 June 2017, of PubMed, Embase, Google Scholar, Cochrane Libraries, China Biology Medicine disc (CBMdisc), Chinese Scientific Journals Fulltext Database (CQVIP), China National Knowledge Infrastructure (CNKI), and Wanfang Database.

The following search terms were used and combined: “(assisted reproductive technology OR ART OR assisted conception OR assisted reproduction OR *in vitro* fertilization OR IVF OR test tube baby OR intracytoplasmic sperm injection OR ICSI OR artificial insemination OR intrauterine insemination OR IU OR cervical canal insemination) AND (congenital malformation OR abnormalities OR birth defect OR defect OR adverse outcomes OR obstetric outcomes OR neonatal outcomes OR perinatal outcomes OR maternal outcomes OR poor outcomes OR pregnancy outcomes OR birth outcomes OR perinatal mortality OR perinatal morbidity) AND (cohort studies OR prospective studies OR follow-up studies).” In addition, we reviewed the reference lists of retrieved articles and recent reviews. Grey literatures (generally refer to non-publicly published literatures) and conference abstracts were not searched. We did not contact authors of the primary studies for additional information.

### Outcome measures

In this study, the singleton pregnancies created with ART were defined as the exposed group, and SC singleton pregnancies as the unexposed group. We defined ART as being conceived by ICSI and/or IVF. The unexposed group included babies born to women who conceived naturally and that in some studies this group may be contaminated by births resulting from ovulation induction (OI) and/or intrauterine insemination (IUI) treatment if they could not be identified and excluded. The main outcome measures for the present study were CMs that were defined as abnormalities that were probably of prenatal origin, including structural, chromosomal, and genetic defects. These malformations were classified according to organs and systems classification based on the World Health Organization International Classification of Diseases (ICD-10), which included abnormalities of the eye, ear, face and neck, respiratory, musculoskeletal, urogenital, digestive, circulatory, and nervous system, cleft lip and/or palate, and those that are chromosomal genetic. Because variations in the definition of malformations exist across countries and cultures, it is extremely difficult to define uniform standards. The early literatures did not always define birth outcomes and in such cases we relied on the outcome terminology in the original articles.

### Study selection

We first performed an initial screening of titles or abstracts. A second screening was based on full-text review. Studies were considered eligible if they**:** 1) were published in Chinese or English; 2) had a prospective or retrospective cohort design; 3) compared the risk of specific CMs of ART singleton pregnancies with those conceived spontaneously; 4) had use of IVF and/or ICSI as the exposure of interest; 5) had use of specific CMs as outcomes of interest; and 6) reported relative risks (RRs) and odd ratios (ORs), with corresponding 95% confidence intervals (CIs) (or data to calculate them). We excluded review papers, non-peer-reviewed local and/or government reports, conference abstracts and presentations. Multiple studies from the same center and/or authors were analyzed to determine whether the most recent publication was an accumulation which included cases reported in earlier publications. If this was evident from our review, then we used only the most recent publication. Additionally, we assessed potential studies to ensure that there was no duplication of case series.

### Data extraction

Data extraction was performed using a standardized data-collection form. We extracted any reported RRs or ORs of specific CMs for ART singleton pregnancies compared with SC singleton pregnancies. Besides, we extracted characteristics for each study. Data were recorded as follows: first author’s name; year of publication; study period; geographic region; sample source (population *vs* clinic-based studies); study design (prospective *vs* retrospective cohort study); sample sizes of ART and SC singleton births, respectively; type of ART; whether patients who achieved a pregnancy with OI and IUI were included in the SC group (yes *vs* no); specific CMs reported (cleft lip and/or palate, chromosomal, eye, ear, face and neck, respiratory, musculoskeletal, urogenital, digestive, circulatory, and nervous system malformations); diagnosis age or time of CMs; confounding factors matched or adjusted (matched or adjusted *vs* crude); and quality score.

### Quality assessment

The quality of each study was evaluated by using the Newcastle-Ottawa Scale(NOS, http://www.ohri.ca/programs/clinical_epidemiology/oxford.asp). In statistics, the scale is a tool used for assessing the quality of non-randomized studies included in a systematic review and/or meta-analysis. Using the tool, each study is judged on 8 items, categorized into 3 groups: the selection of the study groups; the comparability of the groups; and the ascertainment of outcome of interest for cohort studies. Stars awarded for each quality item serve as a quick visual assessment. Stars are awarded such that the highest-quality studies can be awarded as many as 9 stars. If a study gains ≥7 stars, it will be considered of higher methodologic quality. Two reviewers (JBQ and TBY) independently conducted the study selection, data extraction, and quality assessment. Any disagreements were resolved through discussion among the authors until consensus was reached.

### Statistical analysis

The OR was used as a common measure of the association between ART singleton pregnancies and risk of specific CMs across studies. The combined ORs and the corresponding 95% CIs were calculated using either fixed-effects models or, in the presence of heterogeneity, random-effects models [37]. Homogeneity of ORs across studies was tested by using the Q statistic (significance level at *P* < 0.10). The *I*^2^ statistic (significance level at *I*^2^ > 50%), which is a quantitative measure of inconsistency across studies, was also calculated [38–39].

Sensitivity analysis was conducted to explore possible explanations for heterogeneity and examine the influence of various exclusion criteria on the overall risk estimate. We also investigated the influence of a single study on the overall risk estimate by omitting 1 study in each turn. Subgroup analysis was performed based on: whether the confounding factors were adjusted or matched, geographic region, sample source, quality score, type of ART, whether patients who achieved a pregnancy with OI and IUI were included in the SC group, study design, diagnostic age or time of CMs, and plurality, to identify potential heterogeneity moderators.

Potential publication bias was assessed by using Begg’s funnel plots and Egger’s linear regression test [40]. Egger’s linear regression test was performed by using SAS version 8.2 (SAS Institute, Cary, NC, USA). Other analyses were performed by Review Manager-version 5.0 (The Nordic Cochrane Centre, The Cochrane Collaboration). A *P* value < 0.05 was considered statistically significant, except where otherwise specified.

## SUPPLEMENTARY MATERIALS FIGURE AND TABLES




