# Supplementary material for: Which type of congenital malformations is significantly increased in singleton pregnancies following after in vitro fertilization/intracytoplasmic sperm injection: a systematic review and meta-analysis
Source: Oncotarget. 2017 Dec 25;9(3):4267–78. doi: 10.18632/oncotarget.23689 (PMC5790538; doi:10.18632/oncotarget.23689)
Supplement: Supplementary file 1 [file oncotarget-09-4267-s001.pdf]

Which type of congenital malformations is significantly increased in singleton pregnancies following after *in vitro* fertilization/intracytoplasmic sperm injection: a systematic review and meta-analysis

SUPPLEMENTARY MATERIALS

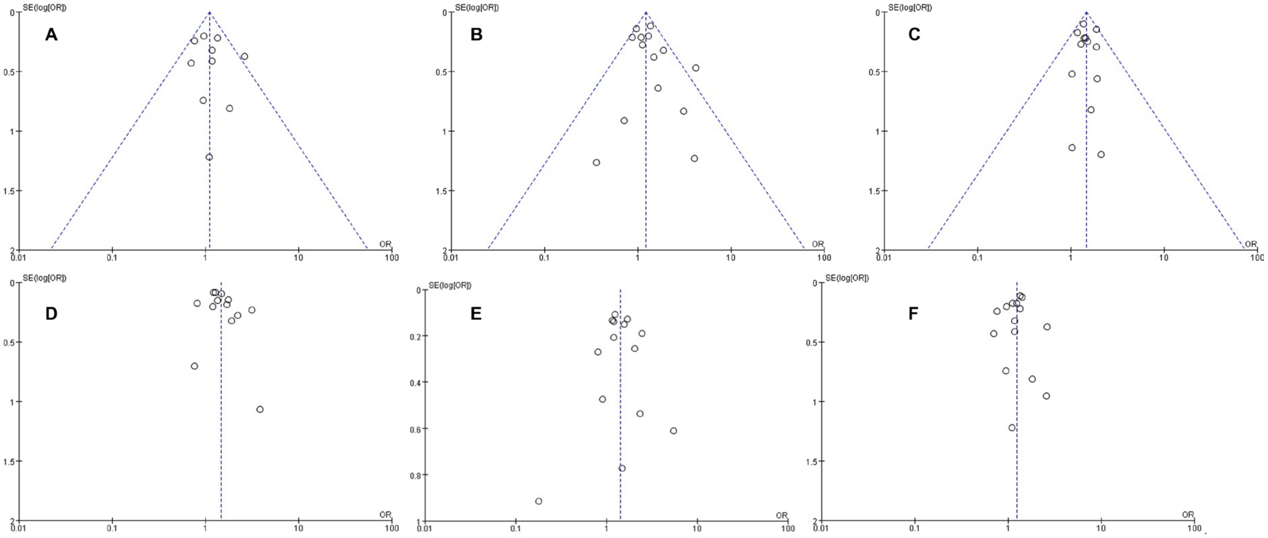

Supplementary Figure 1: Funnel plots of meta-analyses of primary outcomes with at least ten studies (**A** = nervous system malformations; **B** = chromosomal defects; **C** = digestive system malformations; **D** = musculoskeletal system malformations; **E** = urogenital system malformations; **F** = circulatory system malformations).

Supplementary Table 1: Total number of ART and SC singleton births for each specific CM

Specific CM according to organs and systems classification	No. of studies (*n*)	ART singleton births		SC singleton births	
		CM (*n*)	Total	CM (*n*)	Total
Cleft lip and/or palate	4	51	47664	3938	4884340
Eye, ear, face and neck	8	245	79303	998	543123
Nervous system	10	135	85344	2704	1047352
Chromosomal defects	14	400	118541	9003	5058758
Respiratory system	8	100	82659	716	837805
Digestive system	13	374	120490	7072	5470144
Musculoskeletal system	13	1411	93835	10934	1069828
Urogenital system	14	829	95479	10955	1069695
Circulatory system	15	970	127747	13570	5491604

Abbreviations: ART, assisted reproductive technology; SC, spontaneously conceived; CM, congenital malformations

Supplementary Table 2: Subgroup analysis for all specific malformations in singleton pregnancies. See Supplementary_ Table_2
