# Supplementary material for: Which type of congenital malformations is significantly increased in singleton pregnancies following after in vitro fertilization/intracytoplasmic sperm injection: a systematic review and meta-analysis
Source: Oncotarget. 2017 Dec 25;9(3):4267–78. doi: 10.18632/oncotarget.23689 (PMC5790538; doi:10.18632/oncotarget.23689)
Supplement: Supplementary file 2 [file oncotarget-09-4267-s002.docx]

**Supplementary Table 2: Subgroup analysis for all specific malformations in singleton pregnancies**

| **Subgroup variables** | **Cleft lip and/or palate(n=4)**  1.34(1.07-1.69)  **χ^2^=1.80, *P*=0.62; I^2^=0%** | **Eye, ear, face and neck(n=8)**  1.20(1.04-1.39)  **χ^2^=8.23, *P*=0.31; I^2^=15%** | **Nervous system(n=10)**  1.10(0.89-1.35)  **χ^2^=10.35, *P*=0.32; I^2^=13%** | **Chromosomal defects(n=14)**  1.23(1.07-1.40)  **χ^2^=18.99, *P*=0.12; I^2^=32%** | **Respiratory system(n=8)**  1.28(1.01-1.64)  **χ^2^=11.15, *P*=0.13; I^2^=37%** | **Digestive system(n=13)**  1.46(1.29-1.65)  **χ^2^=6.63, *P*=0.88; I^2^=0%** | **Musculoskeletal system(n=13)**  1.47(1.25-1.72)  **χ^2^=33.68, *P*=0.0008; I^2^=64%** | **Urogenital system(n=14)**  1.43(1.18-1.72)  **χ^2^=34.23, *P*=0.001; I^2^=62%** | **Circulatory system(n=15)**  1.39(1.23-1.58)  **χ^2^=26.11, *P*=0.03; I^2^=46%** |
| --- | --- | --- | --- | --- | --- | --- | --- | --- | --- |
| **Adjusted/matcher versus crude data** | **TSD: χ^2^=1.55, *P*=0.21; I^2^=35.6%** | **TSD: χ^2^=0.58, *P*=0.45; I^2^=0%** | **TSD: χ^2^=0.51, *P*=0.47; I^2^=0%** | **TSD: χ^2^=1.38, *P*=0.24; I^2^=27.5%** | **TSD: χ^2^=4.59, *P*=0.03; I^2^=78.2%** | **TSD: χ^2^=0.34, *P*=0.56; I^2^=0%** | **TSD: χ^2^=0.22, *P*=0.64; I^2^=0%** | **TSD: χ^2^=1.71, *P*=0.19; I^2^=41.5%** | **TSD: χ^2^=0.00, *P*=0.97; I^2^=0%** |
| Adjusted/matched | 1.27(0.99-1.62)(n=3)  χ^2^=0.24, *P*=0.88; I^2^=0% | 1.34(0.97-1.85)(n=4)  χ^2^=2.98, *P*=0.39; I^2^=0% | 1.05(0.78-1.41)(n=5)  χ^2^=3.00, *P*=0.56; I^2^=0% | 1.18(1.01-1.36)(n=10)  χ^2^=11.51, *P*=0.24; I^2^=22% | 0.52(0.22-1.20)(n=4)  χ^2^=5.44, *P*=0.14; I^2^=45% | 1.53(1.27-1.84)(n=8)  χ^2^=4.82, *P*=0.68; I^2^=0% | 1.45(1.15-1.84)(n=8)  χ^2^=16.97, *P*=0.02; I^2^=59% | 1.29(0.98-1.69)(n=9)  χ^2^=20.07, *P*=0.01; I^2^=60% | 1.42(1.13-1.78)(n=10)  χ^2^=22.31, *P*=0.008; I^2^=60% |
| Crude | 1.94(1.04-3.62)(n=1)  not applicable | 1.16(0.97-1.39)(n=3)  χ^2^=4.66, *P*=0.10; I^2^=57% | 1.22(0.90-1.66)(n=4)  χ^2^=5.69, *P*=0.13; I^2^=47% | 1.42(1.07-1.89)(n=4)  χ^2^=6.10, *P*=0.11; I^2^=51% | 1.36(1.04-1.78)(n=3)  χ^2^=0.53, *P*=0.77; I^2^=0% | 1.41(1.18-1.69)(n=4)  χ^2^=1.43, *P*=0.70; I^2^=0% | 1.59(1.18-2.13)(n=4)  χ^2^=15.77, *P*=0.001; I^2^=81% | 1.71(1.24-2.35)(n=4)  χ^2^=10.08, *P*=0.02; I^2^=70% | 1.42(1.26-1.59)(n=4)  χ^2^=2.05, *P*=0.56; I^2^=0% |
| **Geographic region** | **TSD: χ^2^=1.77, *P*=0.18; I^2^=43.4%** | **TSD: χ^2^=2.54, *P*=0.28; I^2^=21.1%** | **TSD: χ^2^=3.52, *P*=0.32; I^2^=14.8%** | **TSD: χ^2^=3.74, *P*=0.29; I^2^=19.9%** | **TSD: χ^2^=9.64, *P*=0.02; I^2^=68.9 %** | **TSD: χ^2^=4.33, *P*=0.23; I^2^=30.7%** | **TSD: χ^2^=11.33, *P*=0.01; I^2^=73.5 %** | **TSD: χ^2^=5.89, *P*=0.12; I^2^=49.1%** | **TSD: χ^2^=14.52, *P*=0.002; I^2^=79.3%** |
| Asia | not applicable | 1.09(0.73-1.63)(n=1)  not applicable | 0.76(0.47-1.23)(n=1)  not applicable | 0.97(0.73-1.29)(n=1)  not applicable | 0.33(0.08-1.34)(n=1)  not applicable | 1.29(0.75-2.22)(n=1)  not applicable | 0.82(0.58-1.16)(n=1)  not applicable | 0.80(0.47-1.36)(n=1)  not applicable | 0.91(0.73-1.13)(n=1)  not applicable |
| Europe | 1.96(1.07-3.59)(n=2)  χ^2^=0.02, *P*=0.89; I^2^=0% | 1.18(0.98-1.41)(n=5)  χ^2^=5.69, *P*=0.22; I^2^=30% | 1..20(0.88-1.64)(n=4)  χ^2^=5.44, *P*=0.14; I^2^=45% | 1.31(0.98-1.75)(n=6)  χ^2^=3.30, *P*=0.65; I^2^=0% | 1.37(1.05-1.79)(n=4)  χ^2^=0.92, *P*=0.82; I^2^=0% | 1.43(1.20-1.71)(n=5)  χ^2^=1.19, *P*=0.88; I^2^=0% | 1.33(1.16-1.53)(n=5)  χ^2^=4.75, *P*=0.31; I^2^=16% | 1.70(1.14-2.54)(n=7)  χ^2^=19.43, *P*=0.003; I^2^=69% | 1.41(1.26-1.58)(n=6)  χ^2^=1.28, *P*=0.94; I^2^=0% |
| Oceania | not applicable | not applicable | 1.33(0.91-1.94)(n=3)  χ^2^=0.24, *P*=0.89; I^2^=0% | 1.22(0.94-1.59)(n=4)  χ^2^=11.36, *P*=0.01; I^2^=74% | 0.36(0.11-1.18)(n=1)  not applicable | 1.26(0.96-1.66)(n=3)  χ^2^=0.79, *P*=0.67; I^2^=0% | 1.74(1.32-2.29)(n=4)  χ^2^=10.11, *P*=0.02; I^2^=70% | 1.42(1.14-1.77)(n=4)  χ^2^=7.19, *P*=0.07; I^2^=58% | 1.40(1.18-1.67)(n=4)  χ^2^=2.69, *P*=0.44; I^2^=0% |
| North America | 1.26(0.98-1.62)(n=2)  χ^2^=0.01, *P*=0.91; I^2^=0% | 1.87(1.05-3.33)(n=1)  not applicable | 0.95(0.22-4.10)(n=1)  not applicable | 1.37(1.09-1.73)(n=3)  χ^2^=0.58, *P*=0.75; I^2^=0% | 7.13(0.45-114.05)(n=1)  not applicable | 1.87(1.41-2.48)(n=3)  χ^2^=0.28 *P*=0.87; I^2^=0% | 1.58(0.59-4.23)(n=2)  χ^2^=2.00, *P*=0.16; I^2^=50% | 0.91(0.36-2.30)(n=1)  not applicable | 2.21(1.07-4.56)(n=3)  χ^2^=7.79, *P*=0.02; I^2^=74% |
| **Sample source** | **not applicable** | **TSD: χ^2^=2.42, *P*=0.12; I^2^=58.7%** | **TSD: χ^2^=0.05, *P*=0.82; I^2^=0%** | **TSD: χ^2^=2.79, *P*=0.10; I^2^=64.1%** | **TSD: χ^2^=1.53, *P*=0.22; I^2^=34.8%** | **TSD: χ^2^=0.08, *P*=0.78; I^2^=0%** | **TSD: χ^2^=3.16, *P*=0.08; I^2^=68.4%** | **TSD: χ^2^=0.68, *P*=0.41; I^2^=0%** | **TSD: χ^2^=9.74, *P*=0.002; I^2^=89.7%** |
| Population-based studies | 1.34(1.07-1.69)(n=4)  χ^2^=1.80, *P*=0.62; I^2^=0% | 1.16(0.98-1.37)(n=6)  χ^2^=5.80, *P*=0.33; I^2^=14% | 1.12(0.91-1.39)(n=7)  χ^2^=8.79, *P*=0.19; I^2^=32% | 1.20(1.05-1.37)(n=10)  χ^2^=10.62, *P*=0.30; I^2^=15% | 1.23(0.95-1.58)(n=6)  χ^2^=9.02, *P*=0.11; I^2^=45% | 1.47(1.29-1.68)(n=9)  χ^2^=5.83, *P*=0.67; I^2^=0% | 1.37(1.19-1.58)(n=9)  χ^2^=17.03, *P*=0.03; I^2^=53% | 1.47(1.20-1.80)(n=10)  χ^2^=25.69, *P*=0.002; I^2^=65% | 1.31(1.17-1.46)(n=11)  χ^2^=14.07, *P*=0.17; I^2^=29% |
| Clinic-based studies | not applicable | 1.87(1.05-3.33)(n=1)  not applicable | 1.28(0.44-3.77)(n=2)  χ^2^=0.35, *P*=0.55; I^2^=0% | 2.13(1.10-4.14)(n=4)  χ^2^=5.58, *P*=0.13; I^2^=46% | 7.13(0.45-114.05)(n=1)  not applicable | 1.33(0.65-2.71)(n=3)  χ^2^=0.69, *P*=0.71; I^2^=0% | 2.29(1.32-3.96)(n=3)  χ^2^=4.00, *P*=0.14; I^2^=50% | 0.91(0.30-2.76)(n=3)  χ^2^=7.89, *P*=0.02; I^2^=75% | 2.66(1.73-4.10)(n=3)  χ^2^=2.00, *P*=0.37; I^2^=0% |
| **Quality score** | **not applicable** | **not applicable** | **not applicable** | **TSD: χ^2^=0.94, *P*=0.33; I^2^=0%** | **TSD: χ^2^=1.77, *P*=0.18; I^2^=43.4%** | **not applicable** | **not applicable** | **TSD: χ^2^=5.26, *P*=0.02; I^2^=81.0%** | **not applicable** |
| High | 1.34(1.07-1.69)(n=4)  χ^2^=1.80, *P*=0.62; I^2^=0% | 1.20(1.03-1.41)(n=7)  χ^2^=8.23, *P*=0.22; I^2^=27% | 1.13(0.91-1.40)(n=9)  χ^2^=9.20, *P*=0.33; I^2^=13% | 1.23(1.08-1.40)(n=13)  χ^2^=18.05, *P*=0.11; I^2^=34% | 1.24(0.96-1.61)(n=7)  χ^2^=10.56, *P*=0.10; I^2^=43% | 1.47(1.29-1.67)(n=12)  χ^2^=6.60, *P*=0.83; I^2^=0% | 1.50(1.26-1.78)(n=12)  χ^2^=33.16, *P*=0.0005; I^2^=67% | 1.48(1.22-1.80)(n=12)  χ^2^=28.55, *P*=0.003; I^2^=61% | 1.40(1.22-1.61)(n=14)  χ^2^=26.05, *P*=0.02; I^2^=50% |
| Low | not applicable | not applicable | not applicable | 0.36(0.03-4.32)(n=1)  not applicable | not applicable | not applicable | not applicable | 0.18(0.03-1.08)(n=1)  not applicable | not applicable |
| **Type of ART** | **not applicable** | **TSD: χ^2^=1.85, *P*=0.17; I^2^=46.0%** | **TSD: χ^2^=0.10, *P*=0.75; I^2^=0%** | **TSD: χ^2^=1.31, *P*=0.25; I^2^=23.4%** | **TSD: χ^2^=2.23, *P*=0.14; I^2^=55.1%** | **TSD: χ^2^=0.83, *P*=0.36; I^2^=0%** | **TSD: χ^2^=0.98, *P*=0.32; I^2^=0%** | **TSD: χ^2^=0.69, *P*=0.40; I^2^=0%** | **TSD: χ^2^=0.07, *P*=0.79; I^2^=0%** |
| IVF | 1.96(1.07-3.59)(n=2)  χ^2^=0.02, *P*=0.89; I^2^=0% | 1.34(1.08-1.65)(n=6)  χ^2^=6.55, *P*=0.26; I^2^=24% | 1.19(0.82-1.73)(n=6)  χ^2^=7.86, *P*=0.16; I^2^=36% | 1.18(0.88-1.58)(n=8)  χ^2^=5.13, *P*=0.64; I^2^=0% | 1.16(0.82-1.66)(n=5)  χ^2^=3.64, *P*=0.46; I^2^=0% | 1.51(1.22-1.87)(n=7)  χ^2^=3.00, *P*=0.81; I^2^=0% | 1.60(1.25-2.04)(n=8)  χ^2^=23.37, *P*=0.001; I^2^=70% | 1.47(1.11-1.96)(n=9)  χ^2^=21.96, *P*=0.005; I^2^=64% | 1.41(1.24-1.61)(n=8)  χ^2^=7.29, *P*=0.40; I^2^=4% |
| ICSI | not applicable | 1.03(0.75-1.41)(n=3)  χ^2^=0.49, *P*=0.78; I^2^=0% | 1.32(0.80-2.16)(n=3)  χ^2^=0.10, *P*=0.95; I^2^=0% | 1.53(1.09-2.16)(n=4)  χ^2^=6.36, *P*=0.10; I^2^=53% | 1.74(1.17-2.60)(n=2)  χ^2^=0.00, *P*=0.95; I^2^=0% | 1.28(0.95-1.72)(n=4)  χ^2^=0.86, *P*=0.83; I^2^=0% | 1.39(1.23-1.57)(n=5)  χ^2^=3.03, *P*=0.55; I^2^=0% | 1.79(1.25-2.55)(n=5)  χ^2^=8.35, *P*=0.08; I^2^=52% | 1.46(1.22-1.73)(n=5)  χ^2^=1.42, *P*=0.84; I^2^=0% |
| **Whether patients who achieved a pregnancy with OI and IUI were included in the SC group?** | **TSD: χ^2^=1.91 *P*=0.34; I^2^=0%** | **TSD: χ^2^=7.53, *P*=0.02; I^2^=73.4%** | **TSD: χ^2^=5.47, *P*=0.06; I^2^=63.4%** | **TSD: χ^2^=1.41, *P*=0.49; I^2^=0%** | **TSD: χ^2^=3.80, *P*=0.15; I^2^=47.3%** | **TSD: χ^2^=2.08, *P*=0.35; I^2^=3.9%** | **TSD: χ^2^=7.90, *P*=0.02; I^2^=74.7%** | **TSD: χ^2^=0.36, *P*=0.84; I^2^=0%** | **TSD: χ^2^=0.38, *P*=0.83; I^2^=0%** |
| Yes | 1.25(0.95-1.64)(n=1)  not applicable | 1.09(0.91-1.32)(n=2)  χ^2^=0.05, *P*=0.83; I^2^=0% | 1.07(0.78-1.45)(n=4)  χ^2^=0.87, *P*=0.83; I^2^=0% | 1.36(1.08-1.72)(n=5)  χ^2^=8.20, *P*=0.08; I^2^=51% | 1.34(1.01-1.77)(n=2)  χ^2^=0.33, *P*=0.57; I^2^=0% | 1.39(1.17-1.65)(n=4)  χ^2^=0.49, *P*=0.92; I^2^=0% | 1.54(1.22-1.94)(n=5)  χ^2^=17.08, *P*=0.002; I^2^=77% | 1.44(1.20-1.74)(n=5)  χ^2^=7.68, *P*=0.10; I^2^=48% | 1.41(1.26-1.58)(n=5)  χ^2^=2.61, *P*=0.62; I^2^=0% |
| No | 1.60(1.04-2.47)(n=3)  χ^2^=0.88, *P*=0.64; I^2^=0% | 2.07(1.36-3.13)(n=3)  χ^2^=0.26, *P*=0.88; I^2^=0% | 1.54(1.07-2.21)(n=4)  χ^2^=2.86, *P*=0.41; I^2^=0% | 1.08(0.77-1.53)(n=4)  χ^2^=3.71, *P*=0.29; I^2^=19% | 1.10(0.54-2.24)(n=4)  χ^2^=6.43, *P*=0.09; I^2^=53% | 1.38(1.04-1.84)(n=4)  χ^2^=2.34, *P*=0.50; I^2^=0% | 1.61(1.37-1.88)(n=4)  χ^2^=2.23, *P*=0.53; I^2^=0% | 1.61(0.99-2.63)(n=4)  χ^2^=11.38, *P*=0.010; I^2^=74% | 1.53(1.19-1.96)(n=4)  χ^2^=4.66, *P*=0.20; I^2^=36% |
| **Study design** | **TSD: χ^2^=1.55, *P*=0.21; I^2^=35.6%** | **TSD: χ^2^=0.45, *P*=0.50; I^2^=0%** | **TSD: χ^2^=0.51, *P*=0.47; I^2^=0%** | **TSD: χ^2^=1.38, *P*=0.24; I^2^=27.5%** | **TSD: χ^2^=4.59, *P*=0.03; I^2^=78.2%** | **TSD: χ^2^=0.31, *P*=0.58; I^2^=0%** | **TSD: χ^2^=0.37, *P*=0.54; I^2^=0%** | **TSD: χ^2^=3.69, *P*=0.05; I^2^=72.9%** | **TSD: χ^2^=0.01, *P*=0.94; I^2^=0%** |
| Retrospective cohort | 1.27(0.99-1.62)(n=3)  χ^2^=0.24, *P*=0.88; I^2^=0% | 1.33(0.96-1.83)(n=3)  χ^2^=2.78, *P*=0.25; I^2^=28% | 1.05(0.78-1.41)(n=5)  χ^2^=3.00, *P*=0.56; I^2^=0% | 1.18(1.01-1.36)(n=10)  χ^2^=11.51, *P*=0.24; I^2^=22% | 0.52(0.22-1.20)(n=4)  χ^2^=5.44, *P*=0.14; I^2^=45% | 1.52(1.26-1.84)(n=7)  χ^2^=4.81, *P*=0.57; I^2^=0% | 1.43(1.13-1.82)(n=7)  χ^2^=16.10, *P*=0.01; I^2^=63% | 1.22(0.96-1.56)(n=8)  χ^2^=14.53, *P*=0.04; I^2^=52% | 1.43(1.13-1.81)(n=9)  χ^2^=22.30, *P*=0.004; I^2^=64% |
| Prospective cohort | 1.94(1.04-3.62)(n=1)  not applicable | 1.17(0.97-1.40)(n=4)  χ^2^=4.99, *P*=0.17; I^2^=40% | 1.22(0.90-1.66)(n=4)  χ^2^=5.69, *P*=0.13; I^2^=47% | 1.42(1.07-1.89)(n=4)  χ^2^=6.10, *P*=0.11; I^2^=51% | 1.36(1.04-1.78)(n=3)  χ^2^=0.53, *P*=0.77; I^2^=0% | 1.42(1.19-1.69)(n=5)  χ^2^=1.47, *P*=0.83; I^2^=0% | 1.61(1.21-2.16)(n=5)  χ^2^=16.73, *P*=0.002; I^2^=76% | 1.85(1.31-2.62)(n=5)  χ^2^=14.03, *P*=0.007; I^2^=71% | 1.42(1.26-1.59)(n=5)  χ^2^=2.06, *P*=0.73; I^2^=0% |
| **Diagnostic age or time of CMs** | **TSD: χ^2^=0.19, *P*=0.66; I^2^=0%** | **TSD: χ^2^=2.60, *P*=0.11; I^2^=61.6%** | **TSD: χ^2^=5.05, *P*=0.02; I^2^=80.2%** | **TSD: χ^2^=2.40, *P*=0.12; I^2^=58.4%** | **TSD: χ^2^=0.31, *P*=0.58; I^2^=0%** | **TSD: χ^2^=0.99, *P*=0.32; I^2^=0%** | **TSD: χ^2^=4.66, *P*=0.03; I^2^=78.5%** | **TSD: χ^2^=2.09, *P*=0.15; I^2^=52.1%** | **TSD: χ^2^=0.22, *P*=0.64; I^2^=0%** |
| Birth | 2.30(0.20-26.45)(n=1)  not applicable | 1.09(0.90-1.33)(n=3)  χ^2^=0.83, *P*=0.66; I^2^=0% | 0.87(0.64-1.19)(n=3)  χ^2^=0.57, *P*=0.75; I^2^=0% | 1.09(0.89-1.33)(n=7)  χ^2^=5.04, *P*=0.54; I^2^=0% | 1.32(0.95-1.82)(n=3)  χ^2^=4.30, *P*=0.12; I^2^=53% | 1.36(1.12-1.65)(n=4)  χ^2^=0.23, *P*=0.97; I^2^=0% | 1.20(0.95-1.53)(n=5)  χ^2^=8.11, *P*=0.09; I^2^=51% | 1.19(0.84-1.68)(n=6)  χ^2^=11.03, *P*=0.05; I^2^=55% | 1.33(0.97-1.82)(n=6)  χ^2^=16.85, *P*=0.005; I^2^=70% |
| >6 months | 1.34(1.06-1.69)(n=3)  χ^2^=1.61, *P*=0.45; I^2^=0% | 1.44(1.10-1.88)(n=4)  χ^2^=4.79, *P*=0.19; I^2^=37% | 1.42(1.06-1.90)(n=6)  χ^2^=3.58, *P*=0.61; I^2^=0% | 1.34(1.13-1.60)(n=7)  χ^2^=11.55, *P*=0.07; I^2^=48% | 1.13(0.75-1.72)(n=4)  χ^2^=5.95, *P*=0.11; I^2^=50% | 1.55(1.31-1.84)(n=8)  χ^2^=5.37, *P*=0.61; I^2^=0% | 1.74(1.38-2.18)(n=7)  χ^2^=19.92, *P*=0.004; I^2^=68% | 1.64(1.26-2.13)(n=7)  χ^2^=19.85, *P*=0.003; I^2^=70% | 1.44(1.28-1.62)(n=8)  χ^2^=6.36, *P*=0.50; I^2^=0% |

Abbreviations: TSD, test for subgroup difference; ART, assisted reproductive technology; SC, spontaneously conceived; ICSI, intracytoplasmic sperm injection; IVF, in vitro fertilization; IUI, intrauterine insemination; OI, ovulation induction; CM, congenital malformations
